# IGF2BP1 regulates chicken skeletal muscle development through an m6A-dependent inter-organ regulatory axis

**DOI:** 10.1016/j.psj.2026.107856

**Published:** 2026-09-28

**Authors:** Xie Wanying, Tian Yixiang, Jia Xintao, Du Wenping, Gao Chaoqun, Lei Yanru, Yang Lan, Wang Kejun, Sun Guirong, Kang Xiangtao, Li Wenting

**Affiliations:** a The Shennong Laboratory, College of Animal Science and Technology, Henan Agricultural University, Zhengzhou 450046, China; b Student Development Center, Zhongyuan Institute of Science and Technology, Xuchang 461000, China; c Key Laboratory of Animal Genetics, Breeding and Reproduction of Shaanxi Province, College of Animal Science and Technology, Northwest A&F University, Yangling 712100, China; d College of Animal Science and Technology, Northeast Agricultural University, Harbin 150030, China; e United Graduate School of Chinese Modern Agriculture, Henan Agricultural University, Zhengzhou 450046, China

**Keywords:** IGF2BP1, Structural variation, m6A modification, CD44, Skeletal muscle

## Abstract

Skeletal muscle development in poultry involves the interplay between local myogenic programs and systemic metabolism, yet the inter-organ regulatory mechanisms linking these processes remain poorly understood. Here, we identified a structural variation (**SV**) in the *IGF2BP1* locus—encoding an m6A reader protein—that promotes chicken muscle development through a liver-to-muscle regulatory axis. In a Gushi × Anka F₂ resource population, chickens carrying the L1 allele exhibited significantly increased muscle mass accompanied by elevated serum cholesterol, triglycerides, and VLDL, indicating enhanced hepatic metabolic output. Integrative multi-omics analyses combining liver transcriptomics, co-immunoprecipitation mass spectrometry (**CoIP-MS**), and RNA immunoprecipitation sequencing (**RIP-seq**) revealed that *IGF2BP1* interacts with proteins enriched in ATP synthesis and lipid metabolism while directly binding *CD44* mRNA. Notably, strong positive correlations between serum lipid levels and muscle *CD44* expression (*r* > 0.90, *P* < 0.01) supported the in vivo relevance of this hepatic-muscular cascade. Mechanistically, *IGF2BP1* stabilized *CD44* transcripts in an m6A-dependent manner, as validated by methylated RNA immunoprecipitation-qPCR (**meRIP-qPCR**), mRNA decay assays, and dual-luciferase reporter experiments. In primary myoblasts, *CD44* overexpression accelerated cell cycle progression and enhanced migratory capacity by 1.84-fold, whereas *CD44* knockdown suppressed both proliferation and migration. These findings define a novel m6A-dependent *IGF2BP1*–*CD44* axis that bridges hepatic metabolism with myogenic programs, providing molecular targets for marker-assisted selection to improve meat production in poultry.

## Introduction

Chickens (*Gallus gallus*) are among the most economically important livestock species worldwide, contributing substantially to global food security and supplying a major source of high-quality animal protein for human consumption (Rubin et al., 2010; Muir et al., 2008; International Chicken Genome Sequencing Consortium, 2004). The growth performance and carcass traits of poultry directly determine the economic returns of the meat industry; among these traits, skeletal muscle mass and quality are the most critical determinants of production efficiency (Ertbjerg and Puolanne, 2017; Velleman, 2007; Feng et al., 2024). Skeletal muscle is the largest tissue by weight in most vertebrates and the primary source of meat in agricultural animals. Consequently, understanding the gene expression regulation that governs skeletal muscle growth is essential for elucidating the phenotypic basis of meat production in livestock (Braun and Gautel, 2011; Shahriyari et al., 2022; Bentzinger et al., 2012). Skeletal muscle development depends on myofiber proliferation and differentiation, which are controlled by integrated genetic and metabolic factors (Yin et al., 2013; Sousa-Victor et al., 2022; Nicholls et al., 2025; Tierney and Sacco, 2016). Unlike mammals, avian species rely almost exclusively on the liver for de novo lipogenesis, making the hepatic-muscular axis a critical determinant of muscle growth (Zaefarian et al., 2019; Trefts et al., 2017; Leclercq 1984; Reshef et al., 2003; Grabner et al., 2021; Wang et al., 2025). Although the genetic architecture of poultry growth has been extensively studied, the inter-tissue regulatory networks that coordinate hepatic metabolic flux with myogenesis remain poorly understood (Andersson, 2001; Goddard and Hayes, 2009; Yu et al., 2025).

*IGF2BP1* encodes a highly conserved RNA-binding protein (**RBP**) that functions as a master post-transcriptional regulator (Elcheva et al., 2008, 2020; Bell et al., 2015). Structurally equipped with K homology (**KH**) domains, *IGF2BP1* acts as a canonical “reader” of N6-methyladenosine (m6A), the most prevalent internal modification in eukaryotic mRNAs (Zaccara et al., 2019; Zhao et al., 2017; Meyer et al., 2012; Dominissini et al., 2012; Linder et al., 2015). By recognizing the m6A consensus sequence, *IGF2BP1* protects target transcripts from degradation and enhances their stability and translation (Huang et al., 2018; Wang et al., 2014, 2015; Roundtree et al., 2017). Our previous pan-genome analysis identified a 3.2-kb deletion in the *IGF2BP1* promoter (GRCg6a chr27: 6,082,202–6,085,435), designated the L1 allele, as the causal variant of a chromosome-27 QTL for body size. Relative to the wild-type W allele, the deletion enhances promoter activity and elevates *IGF2BP1* expression (Wang et al., 2021, 2024; Gao et al., 2025). However, the molecular mechanisms by which this structural variation (SV) drives muscle development and couples hepatic metabolism to myogenic programs remain undefined.

Skeletal muscle development requires metabolic support, supplied by systemic nutrient reallocation (Egan and Zierath, 2013; Karsenty and Olson, 2016; Samuel and Shulman, 2012; Saltiel and Kahn, 2001). In avian species, the liver serves as the exclusive hub for de novo lipogenesis, making hepatic lipid synthesis and very low-density lipoprotein (**VLDL**) secretion the primary nutrient supply chain for peripheral muscle expansion (Brown and Goldstein, 1986; Wang et al., 2021; Sorokin et al., 2023). Translating this hepatic metabolic supply into local structural expansion requires intense extracellular matrix (**ECM**) remodeling and myoblast migration (Feng et al., 2024; Kahane et al., 2002; Kjaer, 2004; Velleman, 1999). *CD44* is a transmembrane glycoprotein that functions as the principal receptor for hyaluronic acid and participates in signaling pathways that regulate cell proliferation, survival, and migration (Ponta et al., 2003; Zoller, 2011; Orian-Rousseau et al., 2002; Naor et al., 1997; Toole, 2004). Upon ligand binding, *CD44* activates receptor tyrosine kinases including EGFR and c-Met, leading to downstream PI3K-Akt and MAPK-ERK signaling that promotes cell cycle progression and inhibits apoptosis (Bourguignon et al., 2010; Toole 2009; Kang et al., 2013). Given these functions, we hypothesized that *CD44* may function as an effector linking metabolic signals to myogenesis through these proliferation-promoting pathways.

Here, we used the “Gushi × Anka” F₂ resource population to characterize the phenotypic impact of the IGF2BP1 structural variation. By integrating cross-tissue transcriptomics, RNA immunoprecipitation sequencing (RIP-seq), and protein interactomics (CoIP-MS), we mapped the m6A-dependent regulatory network regulated by *IGF2BP1*. Our findings define an inter-organ mechanism in which *IGF2BP1* coordinates hepatic metabolism while stabilizing *CD44* transcripts to promote myoblast proliferation and migration. These results provide a framework for understanding how m6A-dependent post-transcriptional regulation contributes to avian muscle development and offer valuable targets for the genetic improvement of meat yield and quality in agricultural animals.

## Materials and methods

### Ethics statement

All animal care and experimental procedures in this study were approved by the Institutional Animal Care and Use Committee of Henan Agricultural University (approval number 11-0085). All experiments were performed in accordance with the Regulations for the Administration of Animal Experiments issued by the Ministry of Agriculture of China.

### Phenotypic measurements

To minimize confounding effects from genetic background variation and to precisely assess the functional impact of the structural variations, intercrossing of L1W/L1W heterozygotes produced a cohort of 296 half-sib or full-sib offspring, including L1L1 (*n*=105), L1W (*n*=134), and WW (*n*=57) genotypes. Genomic DNA was extracted from chicken blood using Qiagen DNeasy Kit. The *IGF2BP1* structural variation was genotyped by PCR amplification of a region flanking the deletion breakpoint. Representative amplicons of each genotype were purified and verified by Sanger sequencing. Primer sequences and PCR conditions are provided in Supplementary Table S1. We then specifically analyzed differences in IGF2BP1 expression and muscle development-related traits between the L1L1 and WW genotypes. Body weight was recorded every 2 weeks. 24 chickens (12 males and 12 females, with each sex comprising 6 L1L1 and 6 WW chickens) were slaughtered at 6 and 12 weeks of age. All birds were fasted for 12h overnight (with free access to water) before blood collection, and samples were collected between 08:00 and 10:00 to minimize prandial and diurnal variation. After slaughter, breast muscle and leg muscle were dissected and weighed on an electronic balance. Muscle length was measured with a digital caliper along the longitudinal axis of the pectoralis major (breast) and tibialis anterior-containing leg muscle bundle. Breast circumference was measured at the widest point of the pectoral region. Muscle fiber diameter was determined from transverse cryosections (10 μm thickness) stained with hematoxylin and eosin (**H&E**). For each muscle sample, a minimum of 6 randomly selected fields of view were captured at 100× magnification. At least 100 individual fibers per sample were measured using the ImageJ software (National Institutes of Health, Bethesda, MD, USA), and the mean fiber diameter was calculated.

### Serum biochemical analysis

Serum concentrations of total cholesterol (**TC**), triglycerides (**TG**), and very low-density lipoprotein (**VLDL**) were assayed using commercially available enzyme-linked immunosorbent assay (**ELISA**) kits (Jiangsu Meimian Industrial Co., Ltd, Jiangsu, China). All measurements were performed in duplicate, and quality control samples were included in each analytical run.

### Cell culture and transfection

Primary chicken hepatocytes were isolated from embryonic day 18 livers by a two-step collagenase perfusion method. Briefly, livers were perfused with Ca²⁺-free Hanks’ balanced salt solution (**HBSS**) followed by 0.05% collagenase type IV (Sigma-Aldrich, St. Louis, MO, USA) solution. Dissociated cells were filtered through a 70-μm cell strainer, washed, and resuspended in William’s E medium supplemented with 10% fetal bovine serum, 1% penicillin-streptomycin, and 10 ng mL⁻¹ EGF. Cells were seeded on collagen-coated plates and cultured at 37°C in a humidified atmosphere containing 5% CO₂. Chicken primary myoblasts were derived from embryonic day 11 leg muscle and cultured in growth medium consisting of DMEM/F-12 (Gibco, USA) supplemented with 10% fetal bovine serum and 1% penicillin-streptomycin.

For overexpression studies, the full-length coding sequences of chicken *IGF2BP1* and *CD44* were amplified and cloned into the pcDNA3.1(+) vector (Invitrogen, USA). For knockdown experiments, small interfering RNAs (siRNAs) targeting *IGF2BP1* (si-*IGF2BP1*), *CD44* (si-*CD44*), and a non-targeting negative control (si-NC) were synthesized by GenePharma (Shanghai, China). Transient transfections were carried out using Lipofectamine 3000 (Thermo Fisher Scientific, USA) following the manufacturer’s recommended protocol. Transfection efficiencies were validated by qRT-PCR 36 h after transfection. Overexpression of *IGF2BP1* in hepatocytes and of *CD44* in myoblasts increased the corresponding mRNA levels by approximately 20-fold and 40-fold, whereas si-*IGF2BP1* and si-*CD44* reduced them to 0.1-fold of the si-NC controls, respectively (Supplementary Fig. S4). These results confirmed the successful overexpression and knockdown of the target genes. The functional assays were performed using freshly isolated, non-passaged primary myoblasts.

### RNA extraction and quantitative real-time PCR (qRT-PCR)

Total RNA was isolated from tissue samples and cultured cells using TRIzol reagent (Invitrogen) per the manufacturer’s instructions. A NanoDrop 2000 spectrophotometer (Thermo Fisher Scientific, Waltham, MA, USA) was employed to evaluate RNA concentration and purity, while agarose gel electrophoresis confirmed integrity. Total RNA (1 μg) was reverse-transcribed into cDNA with the PrimeScript RT Reagent Kit containing gDNA Eraser (TaKaRa, Dalian, China). Amplification reactions were subsequently conducted on a QuantStudio 5 platform using TB Green Premix Ex Taq II (TaKaRa). Each reaction was run in triplicate with the following cycling conditions: 95°C for 30 s, followed by 40 cycles of 95°C for 5 s and 60°C for 30 s. Relative expression levels were calculated using the 2^⁻ΔΔCt^ method (Livak and Schmittgen, 2001) with chicken GAPDH as the internal reference gene.

### Transcriptome sequencing and bioinformatic analysis

RNA-seq was performed on liver tissue samples from the two genotypes and on 3×Flag-IGF2BP1-overexpressing and control myoblasts. Library construction was performed with the NEBNext Ultra II Directional RNA Library Prep Kit, and sequencing was conducted on an Illumina NovaSeq 6000 platform generating 150-bp paired-end reads. Raw reads were filtered to remove adapters and low-quality bases using Trimmomatic. Clean reads were aligned to the chicken reference genome (GRCg6a). Transcript-level quantification was performed with StringTie, and gene-level counts were obtained with featureCounts. Differentially expressed genes (**DEGs**) were identified using the DESeq2 R package with thresholds of log₂(fold change) ≥ 1 and adjusted P-value < 0.05. Gene Ontology (**GO**) and Kyoto Encyclopedia of Genes and Genomes (**KEGG**) pathway enrichment analyses of DEGs were conducted with the clusterProfiler R package. Gene Set Enrichment Analysis (**GSEA**) was additionally performed to capture subtle pathway-level shifts.

### Co-immunoprecipitation-mass spectrometry (CoIP-MS)

For mass spectrometry analysis, primary hepatocyte lysates were subjected to immunoprecipitation using either an anti-3×Flag-IGF2BP1 antibody or isotype-matched IgG as a negative control. The resulting immunocomplexes were sent to GeneCreate (Wuhan, China) for shotgun LC-MS/MS analysis. Briefly, co-immunoprecipitated proteins were enzymatically digested into peptide fragments, which were subsequently resolved by reversed-phase high-performance liquid chromatography and subjected to electrospray ionization tandem mass spectrometry. Peptide ions were detected based on their mass-to-charge ratios, and selected ions underwent collision-induced dissociation to generate fragment ion spectra for sequence identification. Protein composition was determined by database searching against the relevant species-specific protein databases using Mascot or similar search engines.

### RNA immunoprecipitation sequencing (RIP-seq) and RIP-qPCR

RIP was performed using an anti-3×Flag-IGF2BP1 antibody and a Magna RIP RNA-Binding Protein Immunoprecipitation Kit (Millipore, Billerica, MA, USA) according to the manufacturer’s protocol. In brief, 3×Flag-IGF2BP1-overexpressing hepatocytes were lysed in RIP lysis buffer supplemented with RNase inhibitors and protease inhibitors. Cell lysates were incubated with magnetic beads conjugated to anti-IGF2BP1 or control IgG overnight at 4°C. After stringent washing, immunoprecipitated RNA was purified by phenol-chloroform extraction and DNase I treatment. For RIP-seq, libraries were prepared with the NEBNext Ultra II Directional RNA Library Prep Kit for Illumina (New England Biolabs, E7760) and sequenced on an Illumina NovaSeq 6000 platform to generate 150-bp paired-end reads. Sequenced reads were aligned to the chicken genome using STAR, and peak calling was performed with MACS2. Peak distribution across genic features (5′UTR, CDS, 3′UTR) was annotated with ChIPseeker. For RIP-qPCR, immunoprecipitated RNA was reverse-transcribed and analyzed by qRT-PCR as described above. Enrichment was calculated as the percentage of input and normalized to the IgG control.

### m6A motif prediction and methylated RNA immunoprecipitation-qPCR (meRIP-qPCR)

Potential m6A modification sites within the *CD44* coding sequence were predicted using the SRAMP online server (http://www.cuilab.cn/sramp), which integrates sequence-derived features to assign confidence levels (high, moderate, low) to candidate m6A sites. Predicted m6A loci were cross-referenced with RIP-seq peak coordinates to identify overlapping regions, designated as the m6A-dependent CDS regulatory elements of *CD44*.

For meRIP-qPCR, total RNA from liver and leg muscle of L1L1 and WW chickens was fragmented chemically using RNA fragmentation reagents (Invitrogen) at 70°C for 10 min to generate 100–200 nt fragments. Fragmented RNA was immunoprecipitated with an anti-m6A antibody (Synaptic Systems, 202003) or IgG control using the Magna MeRIP m6A Kit (Millipore). Enriched RNA fragments were purified, reverse-transcribed, and analyzed by qRT-PCR using primers flanking the predicted m6A sites within the CD44 CDS region: forward 5′-ATGCAGATGGCACACGTCA-3′, reverse 5′-GGAAAGTCTTTTTCACCTCCTCC-3′. m6A enrichment was expressed as the percentage of input normalized to the IgG control.

### mRNA stability assay

To evaluate *CD44* transcript stability, myoblasts transfected with pcDNA3.1-IGF2BP1, si-IGF2BP1, or their corresponding controls were treated with actinomycin D (ActD; 2 μg mL⁻¹; Sigma-Aldrich) to block nascent transcription. Total RNA was harvested at 0, 10, 30, 60, 180, and 360 min post-ActD addition. *CD44* mRNA levels at each time point were quantified by qRT-PCR and normalized to time-zero values. The mRNA half-life (t₁/₂) was estimated by nonlinear regression assuming first-order decay kinetics using GraphPad Prism.

### Dual-luciferase reporter assay

The *CD44* CDS region containing the predicted m6A motifs (wild-type, CRD-wt) and the corresponding mutant construct in which adenosine residues within the m6A consensus sites were substituted with thymidines (mutant, CRD-mut) were synthesized and cloned into the pmirGLO dual-luciferase reporter vector (Promega, USA) downstream of the firefly luciferase coding sequence. Chicken myoblasts were co-transfected with reporter constructs and pcDNA3.1-IGF2BP1 or empty vector using Lipofectamine 3000. Luciferase activity was quantified 48 h after transfection with the Dual-Luciferase Reporter Assay System (Promega) on a multimode plate reader. Firefly signals were calibrated against Renilla signals, and data are presented as fold change versus the empty-vector control.

### Cell proliferation assays

EdU (5-ethynyl-2′-deoxyuridine) incorporation was performed with the Cell-Light EdU Apollo488 In Vitro Imaging Kit (RiboBio, Guangzhou, China). Briefly, myoblasts transfected with pcDNA3.1-CD44, si-CD44, or controls were incubated with 50 μM EdU for 2 h at 37°C, fixed in 4% paraformaldehyde, permeabilized with 0.5% Triton X-100, and stained with Apollo reaction cocktail. Nuclei were counterstained with DAPI. Images were acquired from 6 random fields per well using a fluorescence microscope (Olympus, Japan), and the percentage of EdU-positive cells was calculated as (EdU⁺/DAPI⁺) × 100% (*n*=3).

For CCK-8 assays, transfected myoblasts were seeded in 96-well plates at a density of 3 × 10³ cells/well. CCK-8 reagent (10 μL; Vazyme Biotech, China) was introduced into each well at 0, 12, 24, 36, and 48 h after seeding, followed by a 2 h incubation at 37°C. The optical density at 450 nm was subsequently recorded on a microplate reader (Thermo Fisher Scientific, USA). Each condition was assessed in 5 technical replicates across 3 independent experiments.

### Cell cycle analysis by flow cytometry

Transfected myoblasts were harvested at 24 h post-transfection, washed twice with cold PBS, and fixed in 70% ethanol at 4°C overnight. Fixed cells were washed, treated with RNase A (100 μg mL⁻¹; Sigma-Aldrich) at 37°C for 30 min, and stained with propidium iodide (PI; 50 μg mL⁻¹; Sigma-Aldrich) for 30 min in the dark. DNA content was analyzed on a BD FACSCanto II flow cytometer. Cell cycle distribution (G0/G1, S, and G2/M phases) was determined using FlowJo software.

### Wound healing (scratch) assay

Transfected myoblasts were grown to approximately 95% confluence in 6-well plates. A uniform linear wound was created by scraping the monolayer with a sterile 200-μL pipette tip, followed by two washes with PBS to remove detached cells. Cells were maintained in serum-reduced medium (2% FBS) to minimize proliferation effects on migration. Wound closure was monitored by phase-contrast microscopy at 0 and 24 h. The percentage of wound closure was calculated as: [(initial scratch area − remaining scratch area) / initial scratch area] × 100%.

### Transwell migration assay

Cell migratory capacity was evaluated using Transwell chambers with 8-μm pore polycarbonate membrane inserts (Corning, Corning, NY, USA) in 24-well plates. Transfected myoblasts (5 × 10⁴ cells) were seeded into the upper chamber in serum-free medium, while the lower chamber contained complete growth medium as a chemoattractant. After incubation for 24 h at 37°C, non-migrated cells on the upper surface were gently removed with a cotton swab. Migrated cells on the lower membrane surface were fixed in 4% paraformaldehyde and stained with 0.1% crystal violet. Images of 6 randomly selected fields per membrane were captured, and migrated cells were counted.

### Statistical analysis

All data are presented as mean ± standard error of the mean (SEM) from at least three independent biological replicates unless otherwise indicated. Statistical comparisons between two groups were performed using Student’s two-tailed unpaired t-test. For comparisons among genotypes, one-way analysis of variance (ANOVA) followed by Tukey’s post hoc test for multiple comparisons was applied. For the in vivo comparisons, sex was balanced within each genotype group (6 L1L1 and 6 WW birds of each sex per time point), and data from males and females were pooled. Normality of data distribution was assessed by the Shapiro-Wilk test, and homogeneity of variance was verified by Levene’s test. When assumptions of parametric testing were not met, the non-parametric Mann-Whitney U or Kruskal-Wallis test was used as an alternative. Correlation analyses were conducted using Pearson’s or Spearman’s rank correlation coefficient as appropriate. Statistical analyses were executed in GraphPad Prism 7.0 (GraphPad Software, USA). A *P*-value < 0.05 was considered statistically significant. Effect sizes were calculated as eta-squared (η²) for one-way ANOVA, and interpreted as small (η² ≥ 0.01), medium (η² ≥ 0.06), and large (η² ≥ 0.14) according to Cohen (1988).

## Results

### The structural variation of IGF2BP1 significantly promotes muscle development traits in chickens

To investigate the role of *IGF2BP1* in chicken muscle development, we first examined the phenotypic differences among *IGF2BP1* genotypes in the “Gushi × Anka” F₂ resource population. The L1L1 genotype consistently exhibited superior muscle development parameters compared with the WW genotype, with L1W chickens generally displaying intermediate values (Fig. 1A). Specifically, L1L1 chickens demonstrated significantly greater leg muscle fiber diameter (**LMFD**; *P* < 0.01), leg muscle length (**LML**; *P* < 0.01), thigh circumference (**TCf**; *P* = 0.05), and leg muscle weight (**LMW**; *P* < 0.01), and breast muscle weight (**BMW**; *P* < 0.01) than WW chickens. Breast muscle fiber diameter (**BMFD**), breast muscle length (**BML**), and breast circumference (**BC**) were also significantly reduced in WW chickens (*P* < 0.05). These results indicate that the L1 allele confers a favorable effect on muscle development in both leg and breast tissues.

**Fig. 1:**
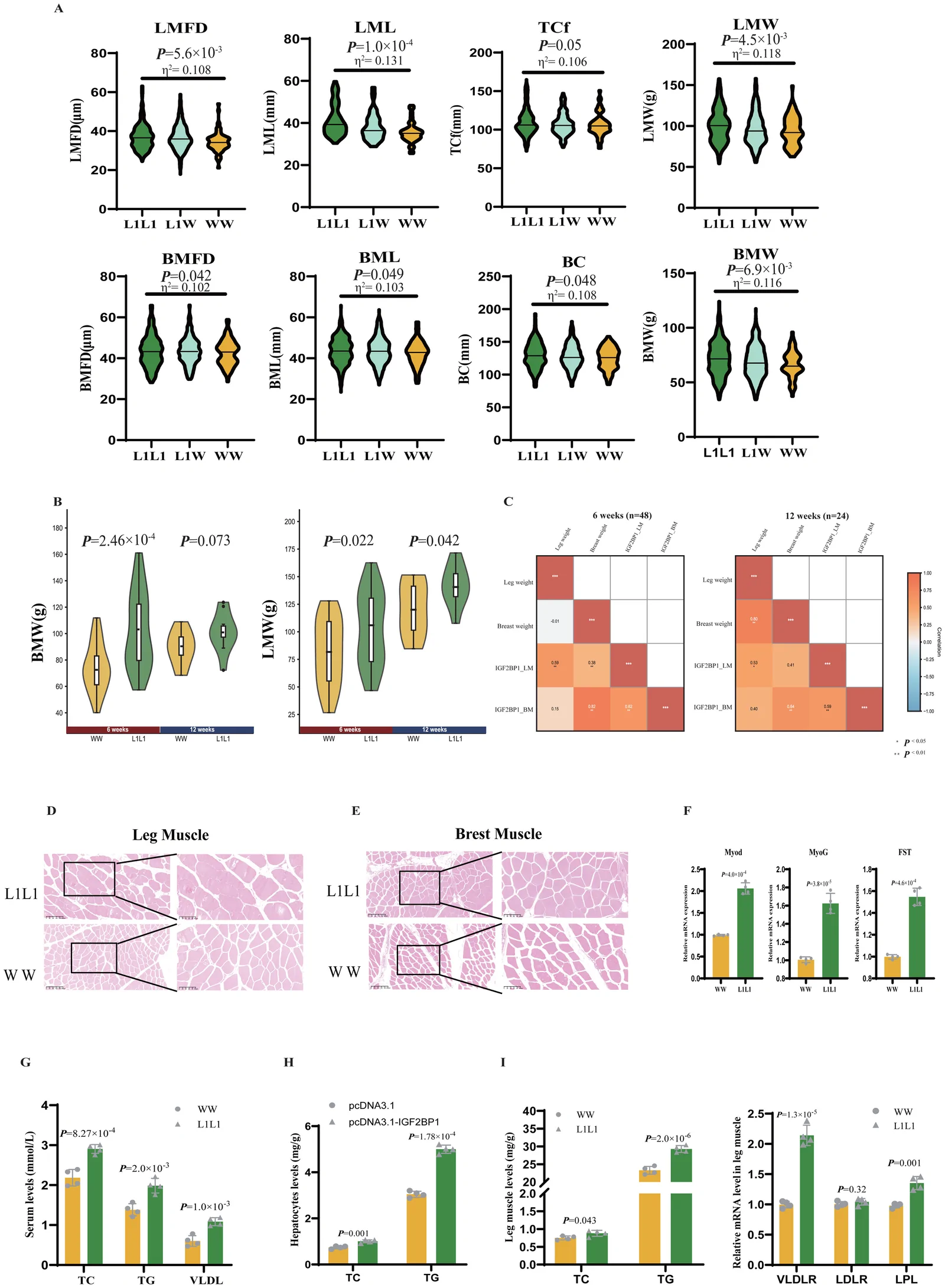
Effects of *IGF2BP1* genotypes on muscle development phenotypes in chickens. (A) Comparisons of muscle development traits, including leg muscle fiber diameter (LMFD), leg muscle length (LML), thigh circumference (TCf), leg muscle weight (LMW), breast muscle fiber diameter (BMFD), breast muscle length (BML), breast circumference (BC), and breast muscle weight (BMW), among different IGF2BP1 genotypes in the Gushi × Anka F2 resource population. (B) The BMW and LMW of full-sib or half-sib offspring with L1L1 and WW genotypes (generated by L1W × L1W crosses) at 6 and 12 weeks of age. (C) Correlation analysis between IGF2BP1 mRNA expression and muscle weights in leg and breast tissues at 6 and 12 weeks of age. (D, E) Representative Hematoxylin and Eosin (H&E) staining images of leg (D) and breast (E) muscle sections from different genotype chickens at 6 weeks. Left panels show overview images (scale bars = 200 μm); right panels show magnified views of the boxed regions (scale bars = 100 μm). (F) Relative mRNA expression of muscle development marker genes in leg muscles determined by qRT-PCR. (G) Serum concentrations of total cholesterol (**TC**), triglyceride (**TG**), and very low-density lipoprotein (**VLDL**) measured by ELISA. (H) TC and TG contents in primary chicken hepatocytes following transfection with pcDNA3.1-IGF2BP1 or the empty vector. (I) Leg muscle TC and TG contents, and relative mRNA levels of very low-density lipoprotein receptor (VLDLR), low-density lipoprotein receptor (LDLR), and lipoprotein lipase (LPL) in leg muscle of WW and L1L1 chickens. Data are presented as means ± SEM. Effect sizes are reported as eta-squared (η²).

To validate these findings and minimize genetic background effects, we compared muscle weights between homozygous L1L1 and WW offspring generated from L1W × L1W crosses. At 6 weeks of age, L1L1 chickens exhibited significantly higher breast muscle weight than WW chickens (*P* = 2.46 × 10⁻⁴), whereas the difference approached but did not reach statistical significance at 12 weeks of age (*P* = 0.073) (Fig. 1B). In leg muscle weight, both developmental stages were significantly greater in L1L1 chickens, suggesting that the promoting effect of the L1 allele on muscle development becomes more pronounced in leg muscle. Consistent with this genotype-dependent growth advantage, longitudinal body weight tracking revealed that L1L1 chickens maintained significantly higher body weight than WW controls throughout the 12-week rearing period (Fig. S1), underscoring the systemic impact of this structural variation on overall somatic growth. Correlation analysis further solidified the genetic causality, revealing a significant positive correlation between IGF2BP1 mRNA expression and muscle weights in both leg and breast tissues across the two developmental stages (Fig. 1C). Histological examination further supported these phenotypic differences, showing that the L1L1 genotype drives striking muscle fiber development, manifesting as expanded cross-sectional areas in both leg and breast muscles compared to the WW genotype (Fig. 1D, E). Concordantly, Oil Red O staining showed increased intramuscular lipid accumulation in both leg and breast muscles of L1L1 chickens compared with WW controls (Fig. S2, S3), suggesting that enhanced hepatic VLDL secretion promotes lipid delivery to peripheral tissues, which may support myofiber expansion. The mRNA expression of core muscle development marker genes was significantly upregulated in the leg muscles of L1L1 individuals (Fig. 1F).

Given that avian muscle growth depends on hepatic lipogenesis and systemic lipid transport, we reasoned that the muscle phenotype of L1L1 chickens might be supported by inter-organ metabolic crosstalk. Consistent with this idea, serum TC, TG and VLDL were all elevated in L1L1 chickens (Fig. 1G), pointing to increased hepatic lipid output. To test this route directly, we first asked whether IGF2BP1 is sufficient to drive lipid production and secretion in hepatocytes. In primary chicken hepatocytes, IGF2BP1 overexpression increased TG and TC within the cells (Fig. 1H), indicating enhanced lipid synthesis. We next examined the receiving tissue in vivo: leg muscle of L1L1 chickens contained more TG and TC than that of WW birds, consistent with the Oil Red O staining (Fig. S2), and showed higher expression of the lipid-uptake genes VLDLR, LPL and LDLR (Fig. 1I). The L1 allele thus appears to couple enhanced hepatic lipid export with increased lipid-uptake capacity in skeletal muscle, which may partly account for the accelerated muscle growth of L1L1 chickens.

### Integrated multi-omics uncovers the hepatic metabolic and inter-tissue interactome driven by IGF2BP1

Avian liver serves as a central metabolic hub. Since the SV of *IGF2BP1* simultaneously influenced muscle development and systemic lipid metabolism, we speculated that *IGF2BP1* may exert its effects on muscle through inter-tissue regulatory mechanisms. We first confirmed that *IGF2BP1* mRNA was significantly higher in the livers of L1L1 chickens (Fig. 2A). To dissect the molecular cascade triggered by this high expression, we performed liver RNA-seq. The volcano plot highlighted a massive transcriptomic shift between L1L1 and WW genotypes (Fig. 2B), with Gene Ontology (**GO**) enrichment indicating that the differentially expressed genes (**DEGs**) were heavily concentrated in energy supply, cytoskeletal dynamics, signal transduction, and cell division (Fig. 2C).

**Fig. 2:**
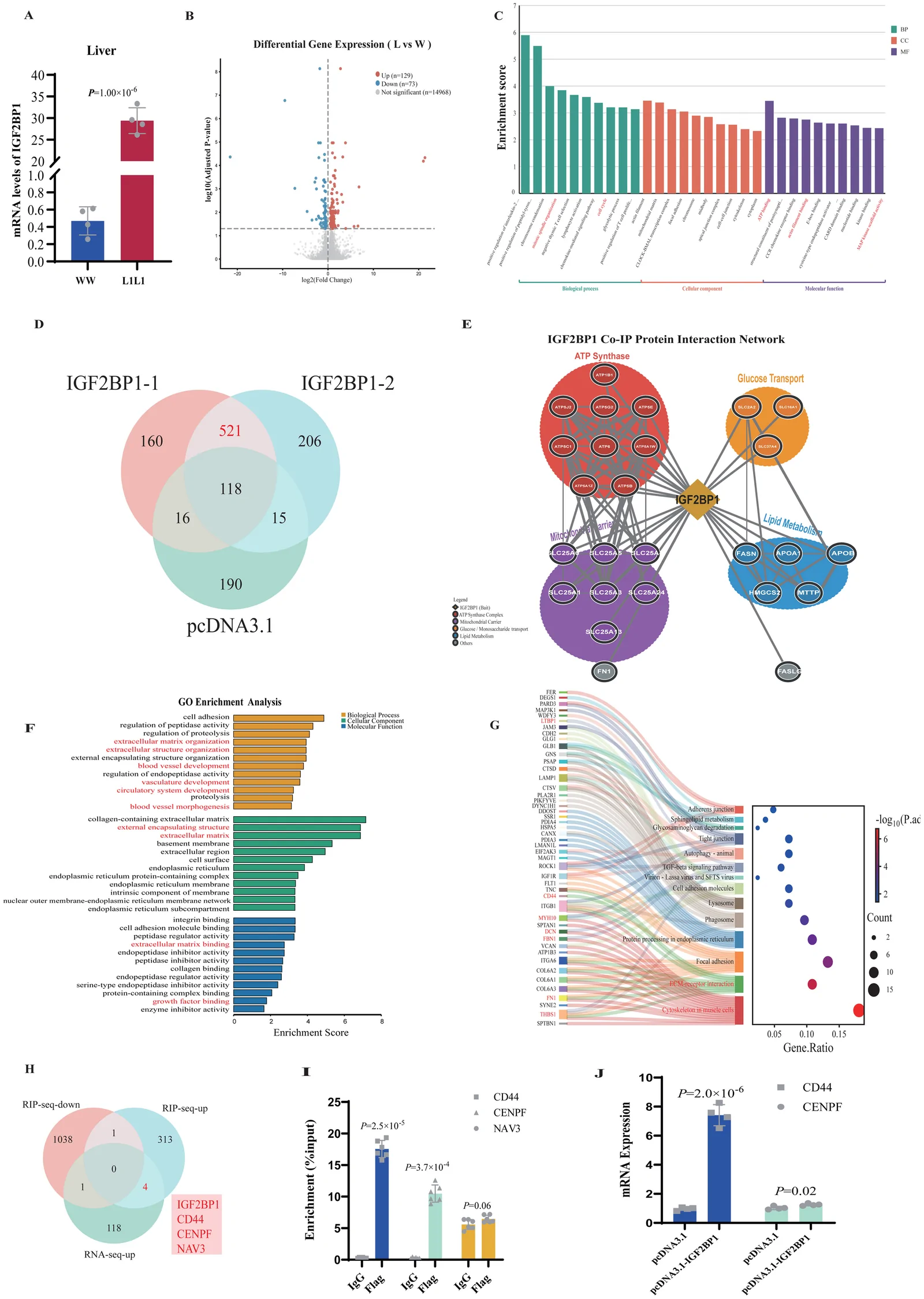
Integrated multi-omics uncovers the hepatic metabolic and inter-tissue interactome driven by *IGF2BP1*. (A) Relative mRNA expression of *IGF2BP1* in the liver of different genotypes (L1L1 vs WW). (B) Volcano plot showing the differentially expressed genes (DEGs) in the liver transcriptome between L1L1 and WW genotypes. (C) Gene Ontology (GO) enrichment analysis of the DEGs identified in the liver transcriptome. (D) Venn diagram illustrating the overlapping and distinct proteins identified by *IGF2BP1*-CoIP coupled with mass spectrometry in primary hepatocytes overexpressing 3×Flag-*IGF2BP1*, with isotype-matched IgG as the negative control. (E) Protein-protein interaction (PPI) network of representative targets selected from the 521 differential proteins, highlighting distinct functional modules (e.g., ATP synthesis, lipid metabolism). (F,G) GO (F) and KEGG (G) enrichment analyses of the targeted transcripts identified by RIP-seq in the IGF2BP1-overexpressing primary hepatocytes. (H) Venn diagram showing the intersection among RIP-seq targets (up- and down-regulated) and the up-regulated DEGs from the liver transcriptome, identifying 4 core overlapping genes. (I) Validation of the specific binding between IGF2BP1 and core target mRNAs via RIP-qPCR. (J) Validation of the relative mRNA expression levels of selected DEGs in the liver using qRT-PCR. Data are presented as means ± SEM.

Recognizing that *IGF2BP1* functions as an RNA-binding protein (**RBP**) that relies on spatial complexes for target regulation, we performed Co-IP coupled with mass spectrometry (**CoIP-MS**) in primary hepatocytes that overexpressed *IGF2BP1*. We identified 521 significantly different proteins interacting with *IGF2BP1* (Fig. 2D). Crucially, the Protein-Protein Interaction (**PPI**) network revealed that these co-precipitated proteins were predominantly enriched in ATP synthesis and lipid metabolism (Fig. 2E), indicating that *IGF2BP1* interacts with a broad spectrum of proteins involved in cellular energy metabolism.

To directly identify the RNA targets of *IGF2BP1*, we conducted RNA immunoprecipitation sequencing (**RIP-seq**) in an *IGF2BP1*-overexpressing primary hepatocytes. GO and KEGG enrichment analyses of the *IGF2BP1*-bound transcripts revealed enrichment in pathways related to growth factor binding, extracellular matrix-receptor interaction, and circulatory system development (Fig. 2F, G), which overlapped substantially with the liver transcriptomic signatures observed between L1L1 and WW genotypes. Integrative analysis of the RIP-seq targets and liver DEGs identified four core genes (*IGF2BP1, CD44, CENPF*, and *NAV3*) that were commonly upregulated across datasets (Fig. 2H). RIP-qPCR validation confirmed that IGF2BP1 specifically bound to *CD44* and *CENPF* mRNAs (Fig. 2I), and qRT-PCR analysis revealed that, among the candidate targets, *CD44* was significantly upregulated in the L1L1 genotype, suggesting that it may represent a core candidate target of *IGF2BP1* in chicken liver (Fig. 2J).

### IGF2BP1 stabilizes CD44 mRNA in an m6A-dependent manner

Having established *CD44* as a principal target of *IGF2BP1* in the liver, we next investigated the molecular basis of this interaction and evaluated its cross-tissue correlation in muscle tissue. Analysis of our RIP-seq datasets revealed distinct *IGF2BP1*-binding peaks spanning the *CD44* transcript. Given the established role of *IGF2BP1* as a canonical m6A reader, we employed the SRAMP algorithm to map potential m6A motifs within the *CD44* coding sequence (**CDS**). This spatial prediction yielded multiple putative m6A loci, two of which mapped precisely to the observed RIP-seq peaks. We designated these intersecting focal points as the coding region determinant (**CRD**) of *CD44* (Fig. 3A). To validate this functional link in vivo, methylated RNA immunoprecipitation-qPCR (**meRIP-qPCR**) demonstrated a significant increase in *CD44* m6A enrichment within both the liver and leg muscle of L1L1 chickens relative to WW controls (Fig. 3B, C), indicating that *IGF2BP1* influences the m6A modification status of *CD44* in liver and leg muscle, a mechanism further corroborated by the significant upregulation of *CD44* mRNA in the corresponding tissues of L1L1 birds (Fig. 3D).

**Fig. 3:**
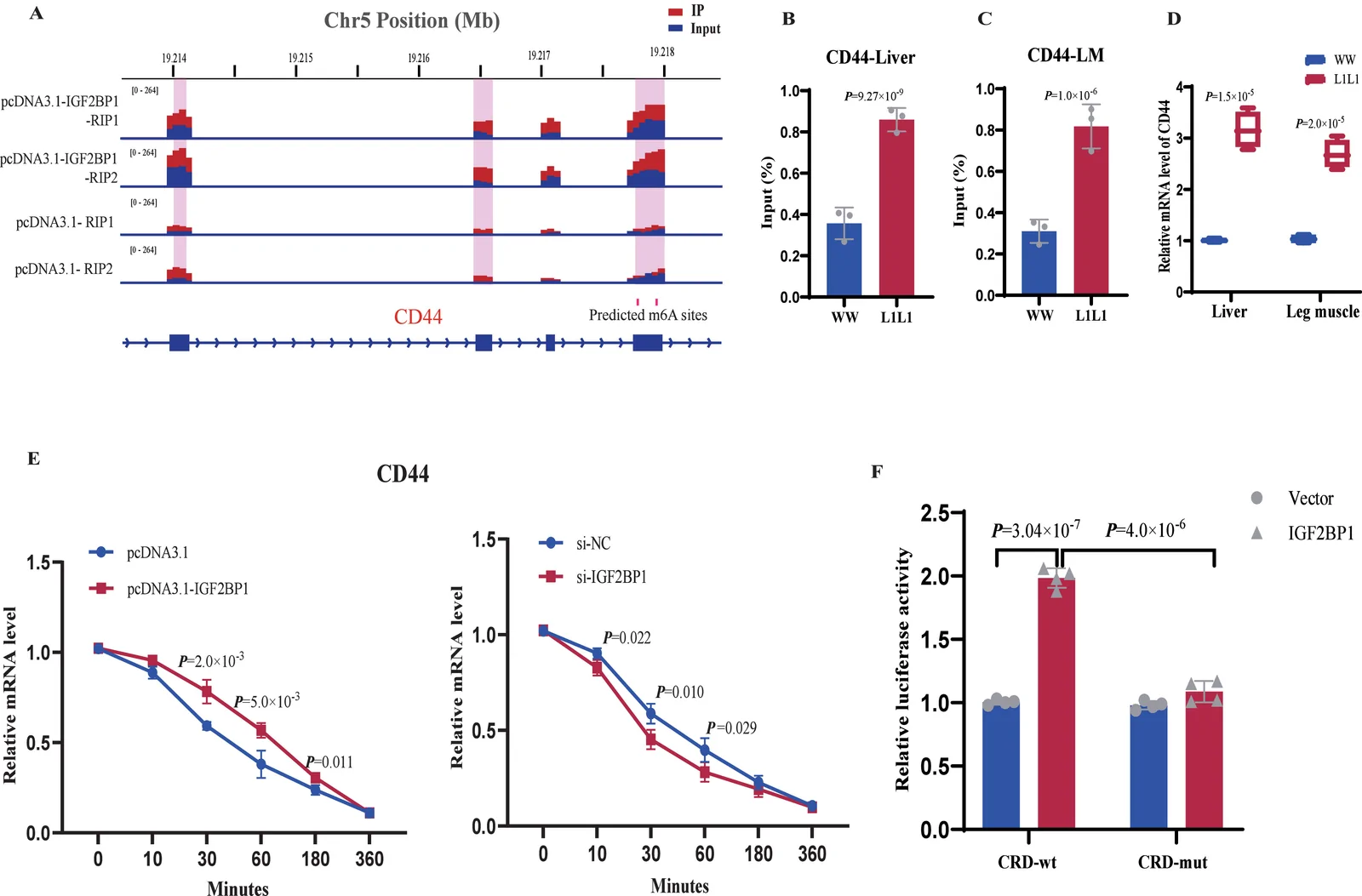
*IGF2BP1* stabilizes *CD44* mRNA in an m6A-dependent manner. (A) Integrative Genomics Viewer (IGV) visualization of RNA immunoprecipitation sequencing (RIP-seq) peaks. The red shading indicates the specific binding of *IGF2BP1* to the *CD44* transcript. (B,C) Methylated RNA immunoprecipitation quantitative PCR (meRIP-qPCR) analysis evaluating the m6A enrichment of *CD44* in the liver (B) and leg muscle (LM) (C) of WW and L1L1 genotype chickens. (D) Relative mRNA expression levels of *CD44* in the liver and leg muscle between WW and L1L1 genotypes determined by qRT-PCR. (E) mRNA stability assay in myoblasts. Cells were transfected with pcDNA3.1-*IGF2BP1* (left) or si-*IGF2BP1* (right) alongside their respective controls, and transcription was halted using actinomycin D (ActD). The degradation rate of *CD44* mRNA was monitored over 360 minutes. (F) Dual-luciferase reporter assay evaluating the interaction between *IGF2BP1* and the coding region determinant (CRD) of *CD44*. Vectors containing the wild-type (CRD-wt) or m6A-mutated (CRD-mut) *CD44* sequences were co-transfected with an *IGF2BP1* overexpression plasmid or an empty vector. Data are presented as means ± SEM.

To determine whether this upregulation stems from post-transcriptional stabilization, we assessed RNA decay kinetics in myoblasts following actinomycin D (**ActD**) treatment. *IGF2BP1* overexpression significantly extended the half-life of *CD44* mRNA, whereas knockdown of *IGF2BP1* markedly accelerated transcript decay over a 360-minute period (Fig. 3E). Furthermore, dual-luciferase reporter assays incorporating the *CD44* coding region determinant (CRD) revealed that the regulatory enhancement conferred by *IGF2BP1* was abrogated upon mutation of the predicted m6A motifs (CRD-mut) (Fig. 3F). Together, these results indicate that *IGF2BP1* stabilizes *CD44* mRNA in an m6A-dependent manner.

### CD44 serves as a conserved inter-tissue target mediating myoblast proliferation and migration

Having identified *CD44* as an m6A-dependent target of *IGF2BP1*, we next investigated its potential role in mediating *IGF2BP1*-related muscle development. It is noteworthy that *NAV3*, which was initially identified as a candidate target through integrative multi-omics analysis (Fig. 2H), failed to validate in RIP-qPCR experiments (Fig. 2I) and was therefore excluded from further functional characterization. Integrative analysis of liver RIP-seq, liver RNA-seq, and *IGF2BP1*-overexpressing myoblast transcriptomes subsequently identified *CD44* as a notable shared target across these datasets (Fig. 4A), reinforcing its status as a core target gene. GO and KEGG enrichment analyses of DEGs in IGF2BP1-overexpressing myoblasts revealed significant enrichment in pathways related to cell proliferation, growth factor receptor binding, and growth factor activity (Fig. 4B, C), phenocopying the functional categories identified in the liver.

**Fig. 4:**
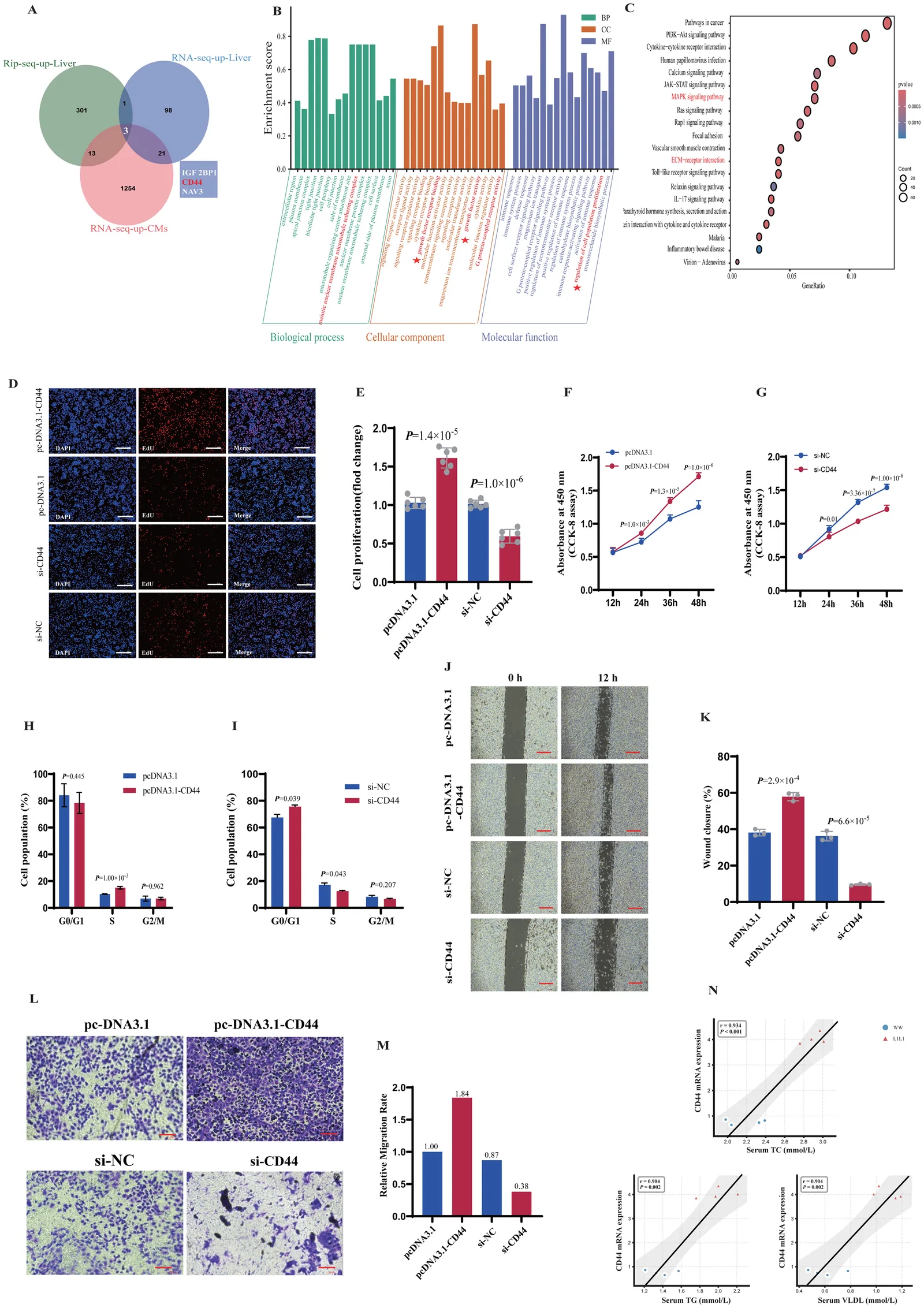
*CD44* acts as a conserved target of *IGF2BP1* to promote myoblast proliferation and migration. (A) Venn diagram showing the overlap among upregulated genes from *IGF2BP1* RIP-seq in liver, upregulated genes from liver RNA-seq (L1L1 vs. WW), and upregulated genes from *IGF2BP1*-overexpressing myoblast RNA-seq. The intersection identified *CD44* as a common target gene across tissues. (B, C) Gene Ontology (GO) (B) and Kyoto Encyclopedia of Genes and Genomes (KEGG) (C) enrichment analyses of the differentially expressed genes (DEGs) in myoblasts overexpressing *IGF2BP1*. (D, E) Representative images and quantification of the 5-ethynyl-2′-deoxyuridine (EdU) incorporation assay, evaluating myoblast proliferation following transfection with *CD44* overexpression plasmid (pcDNA3.1-CD44), small interfering RNA targeting *CD44* (si-*CD44*), or their respective negative controls (scale bars = 100 μm). (F, G) Cell Counting Kit-8 (CCK-8) assays measuring myoblast viability and proliferation rates at continuous time points post-transfection for *CD44* overexpression (F) and knockdown (G). (H, I) Flow cytometry analysis of the cell cycle distribution (G0/G1, S, and G2/M phases) in myoblasts with *CD44* overexpression (H) or knockdown (I). (J–M) Assessment of myoblast migratory capacity using wound healing (scratch) assays (J, K) and Transwell migration assays (L, M) following the modulation of *CD44* expression (scale bars = 100 μm). (N) Correlation between serum lipid indicators and muscle *CD44* mRNA expression in chickens. Pearson correlation analyses were performed using individual data points from WW (blue circles, *n* = 4) and L1L1 (red triangles, *n* = 4) genotype chickens. Data are presented as means ± SEM of at least three independent biological replicates. All functional assays (D-M) were performed using freshly isolated, non-passaged primary myoblasts; EdU incorporation was quantified at 36 h post-transfection, CCK-8 assays at 0-48 h after seeding, and flow cytometry, wound-healing, and Transwell migration assays at 24 h post-transfection.

To directly assess the functional role of *CD44* in muscle cells, we performed a series of in vitro phenotypic assays. Modulation of *CD44* directly dictated myoblast proliferative capacity. EdU incorporation (Fig. 4D, E) and continuous CCK-8 tracking (Fig. 4F, G) showed that CD44 overexpression promoted cell proliferation, whereas its knockdown severely impaired viability. Flow cytometry further revealed that *CD44* overexpression accelerated the cell cycle transition, driving cells from the G0/G1 phase into the active S and G2/M phases, an effect that was reversed by *CD44* knockdown (Fig. 4H, I). Muscle fiber development requires myoblast migration and fusion. Wound healing (Fig. 4J, K) and Transwell migration assays (Fig. 4L) consistently showed that *CD44* overexpression increased myoblast migratory capacity by 1.84-fold, whereas *CD44* knockdown reduced it to 0.38-fold relative to controls (Fig. 4M).

Furthermore, to test whether the hepatic metabolic phenotype of the L1 allele translates to elevated *CD44* expression in the muscle, we analyzed the Pearson correlation between serum lipid indicators and leg muscle *CD44* mRNA levels across both genotypes. All three serum lipid parameters demonstrated strong positive correlations with CD44 expression (Fig. 4N): total cholesterol (TC; *r* = 0.934, *P* < 0.001), triglycerides (TG; *r* = 0.904, *P* = 0.002), and very low-density lipoprotein (VLDL; *r* = 0.904, *P* = 0.002). These physiological associations suggest that the enhanced hepatic lipid output observed in L1L1 chickens is linked to upregulated *CD44* signaling in skeletal muscle, in line with the proposed liver-to-muscle regulatory cascade. The direct measurements of intramuscular lipid accumulation and lipid-uptake gene expression (Fig. 1I) provide complementary experimental support for this link. Collectively, these findings indicate that *CD44* acts as a downstream effector of *IGF2BP1* to promote both proliferation and migration of myoblasts, providing a mechanistic basis for the muscle development phenotype associated with *IGF2BP1*.

## Discussion

Skeletal muscle development has largely been studied as a cell-autonomous process, driven by intrinsic transcriptional programs such as *MyoD* and *Myf5* (Braun and Gautel, 2011; Bentzinger et al., 2012). Our data from chickens point to a role for the liver as well. The *IGF2BP1* structural variation (SV) characterized in this study triggers a coordinated cross-tissue response, coupling enhanced hepatic lipid output with targeted *CD44* mRNA stabilization in skeletal muscle. This synergistic regulation suggests that m6A-mediated post-transcriptional regulation acts as a critical mediator of inter-organ crosstalk beyond the classical endocrine or paracrine routes.

Tissue growth inherently demands a massive, sustained influx of metabolic substrates that local supply cannot meet (Egan and Zierath, 2013; Karsenty and Olson, 2016; Samuel and Shulman, 2012; Saltiel and Kahn, 2001). Avian species are unique in this regard, as their de novo lipogenesis occurs almost entirely in the liver. Hepatic lipid synthesis and subsequent VLDL secretion thus serve as the main nutrient pipeline for peripheral tissues (Zaefarian et al., 2019; Leclercq, 1984; Grabner et al., 2021). The elevated serum cholesterol, triglycerides and VLDL in L1L1 chickens are consistent with increased hepatic lipid output, and these circulating lipids could furnish the fatty acids needed for energy as well as the membrane phospholipids required for myoblast fusion. Functional evidence supports this supply route at both ends: in primary hepatocytes, IGF2BP1 overexpression increased cellular TG and TC, and in L1L1 chickens leg muscle accumulated more TG and TC and expressed higher levels of the lipid-uptake genes *VLDLR, LPL* and *LDLR*, which capture circulating *VLDL* and liberate its fatty-acid cargo at the vascular endothelium. Hepatic lipid export and muscle lipid uptake thus appear to be coordinately enhanced, which is consistent with the proposed inter-organ axis. The identity of the circulating mediator that couples liver-derived lipids to the local myogenic program remains to be determined. To look closer at how *IGF2BP1* drives this hepatic shift, CoIP-MS was performed, which revealed that *IGF2BP1*-interacting proteins were enriched in ATP synthesis and lipid metabolism pathways. RIP-seq analysis further indicated that the genes bound to *IGF2BP1* were mainly enriched in pathways regulating TGF-β signaling, extracellular matrix-receptor interactions, and cytoskeleton dynamics, which are closely related to the coordination of cell growth and tissue remodeling. These results indicate that the *IGF2BP1* structural variation enhances hepatic lipid synthesis and transport, while priming the structural networks necessary to accommodate accelerated peripheral growth.

How does this hepatic metabolic output actually trigger a local myogenic response? We needed to find the precise downstream effectors. *IGF2BP1* is well known to protect target mRNAs from decay by binding to GG(m6A)C consensus sequences via its KH domains (Zaccara et al., 2019; Zhao et al., 2017; Meyer et al., 2012; Dominissini et al., 2012). By integrating RIP-seq with cross-tissue transcriptomics data, CD44 was identified as a primary candidate bridging liver output with local muscle growth. We validated this relationship using meRIP-qPCR and specific mutations in the *CD44* coding region determinant (CRD). The data confirmed that IGF2BP1 must rely on intact m6A motifs to extend the half-life of *CD44* mRNA, underscoring that *CD44* is a specific m6A-dependent target in this context.

*CD44* functions as a widely expressed transmembrane glycoprotein and hyaluronic acid receptor, translating external ECM signals into intracellular changes (Ponta et al., 2003; Zoller, 2011; Orian-Rousseau et al., 2002). Previous work shows it is highly expressed in activated satellite cells, and losing *CD44* severely limits myoblast migration (Mylona et al., 2006). Our in vitro data align perfectly with this. Overexpressing *CD44* nearly doubled the migratory capacity (1.84-fold) of myoblasts and visibly accelerated their cell cycle, both of which are fundamental steps for muscle fiber formation and tissue expansion (Skandalis et al., 2019; Senbanjo and Chellaiah, 2017). The fact that *IGF2BP1* stabilizes *CD44* via m6A binding implies that the *IGF2BP1* structural variation might actually be converting what is normally a short-lived regenerative signal into a continuous pro-growth command. Ultimately, these mechanisms thus provide a molecular basis for the superior muscle traits of L1L1 chickens.

The developmental trajectory of the L1 effect directly underscores its agricultural utility. Phenotypic monitoring reveals that the L1 allele confers a robust growth advantage during the critical rapid growth phase (6-12 weeks). While the pronounced divergence in breast muscle mass at 6 weeks moderately attenuates by week 12, the phenotypic gains in leg muscle and overall body weight are stably maintained (Fig. 1B; Fig. S1). Given that many indigenous/dual-purpose breeds are typically marketed at approximately 14 weeks of age, this persistent growth trajectory translates directly to significant economic value. Importantly, our previous pan-genome survey established that the L1 allele is nearly fixed in modern commercial broilers but remains rare in indigenous populations (Wang et al., 2021). Consequently, while the genetic potential of this structural variation has been thoroughly exploited in commercial broiler breeding, it now presents a precise target for marker-assisted introgression. Introducing the L1 allele offers a validated, directed genetic strategy to enhance the growth rate and meat yield of slower-growing indigenous breeds. (Fig. 5)

**Fig. 5:**
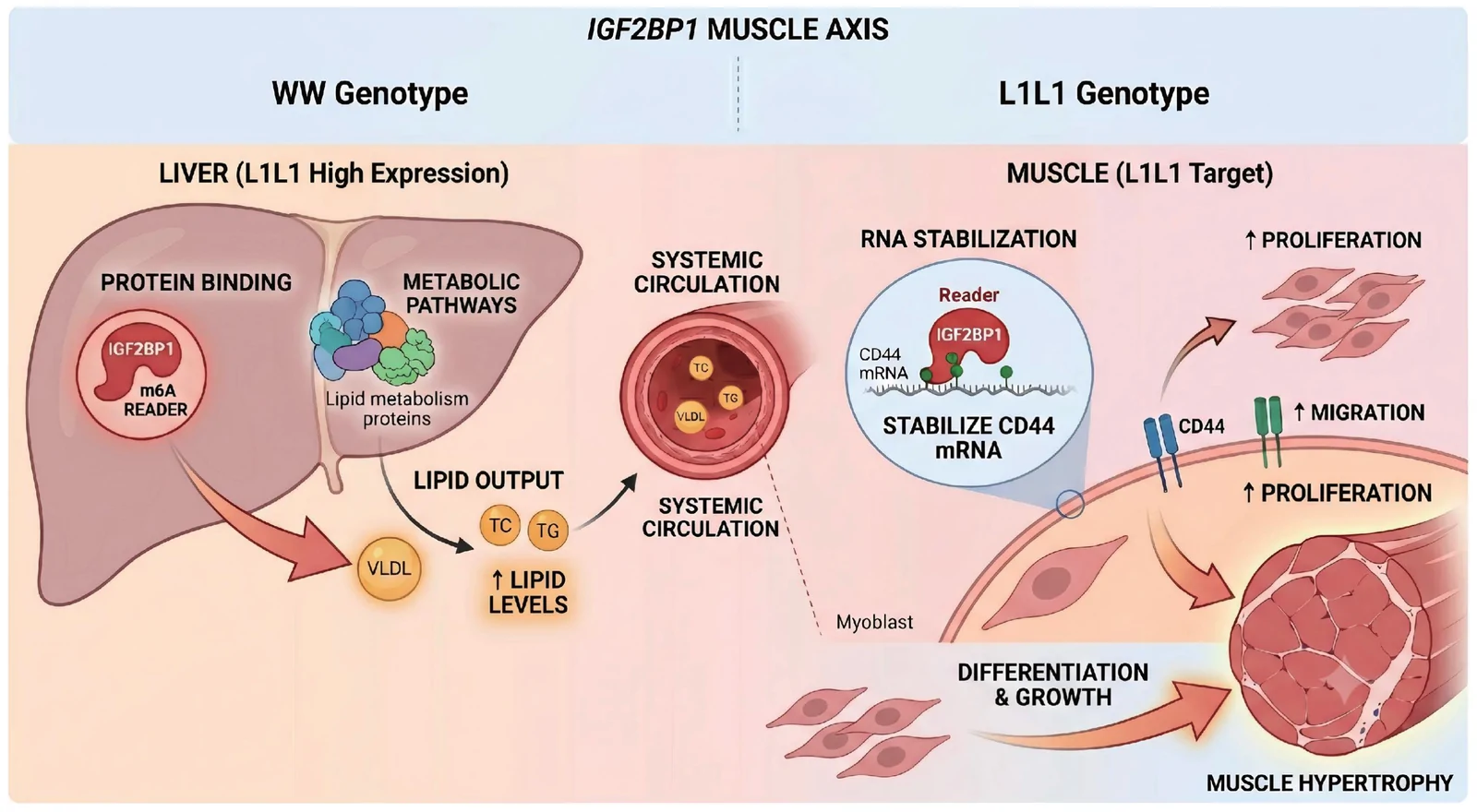
Schematic model of the inter-organ regulatory network. The *IGF2BP1* structural variation (L1 allele) enhances hepatic lipid synthesis and VLDL secretion, increasing systemic lipid supply to skeletal muscle. Concurrently, *IGF2BP1* stabilizes *CD44* mRNA in an m6A-dependent manner, promoting myoblast proliferation and migration. This dual mechanism coordinately drives skeletal muscle development in chickens.

## Conclusions

In this study, we show that a structural variation in the *IGF2BP1* locus influences muscle development in chickens through an inter-tissue mechanism. The *IGF2BP1* SV enhances hepatic lipogenesis and systemic lipid transport, while in muscle it stabilizes *CD44* mRNA in an m6A-dependent manner to promote myoblast proliferation and migration. These findings indicate that post-transcriptional regulation can coordinate metabolic supply between tissues, and that the IGF2BP1-CD44 axis provides practically useful targets for genetic improvement of meat yield in poultry.

## Funding

This study was supported by the National Natural Science Foundation of China (32272866 and 32573209), Natural Science Foundation of Henan Province (262300421123) and the Program for Innovative Research Team at the University of Henan Province (26IRTSTHN015 and 25HASTIT043).

## Author contributions

Wanying Xie and Yixiang Tian: Performed the majority of the experiments, analyzed and interpreted the data, and wrote the original draft. Xintao Jia and Wenping Du: Performed the phenotypic measurements and sample collection, and critically reviewed the manuscript. Chaoqun Gao and Yanru Lei: Designed and performed the cell culture and functional assays, analyzed the in vitro data, and critically reviewed the manuscript. Lan Yang: Performed the bioinformatic analysis, interpreted the results, and critically reviewed the manuscript. Kejun Wang, Guirong Sun, and Xiangtao Kang: Conceived and designed the study, supervised the research, and critically revised the manuscript. Wenting Li: Conceived and designed the project and experiments, supervised the research, and critically revised the manuscript. All authors reviewed and approved the final version of the manuscript and agree to be accountable for all aspects of the work.

## Ethics declaration

This study was conducted in accordance with the ARRIVE (Animal Research: Reporting of In Vivo Experiments) guidelines. This study was approved by the the Institutional Animal Care and Use Committee of Henan Agricultural University. (Approval No. 11-0085)

## Disclosures

The authors declare that they have no known competing financial interests or personal relationships that could have appeared to influence the work reported in this paper.

## Data Availability

The data that support the findings of this study are available from the corresponding author upon reasonable request.

## Footnotes

Supplementary material associated with this article can be found, in the online version, at doi:10.1016/j.psj.2026.107856.
