# Supplementary material for: IGF2BP1 regulates chicken skeletal muscle development through an m6A-dependent inter-organ regulatory axis
Source: Poult Sci. 2026 Sep 28;105(12):107856. doi: 10.1016/j.psj.2026.107856 (PMC13659501; doi:10.1016/j.psj.2026.107856)
Supplement: Supplementary file 1 [file mmc1.docx]

**Fig. S1** Growth curves of body weight from birth to 12 weeks for WW and L1L1 genotype chickens derived from L1W × L1W full-sib or half-sib families. Body weights were recorded every 2 weeks. Statistical significance between genotypes was assessed by unpaired t-test at each time point, with the exact *P* values indicated.

**Fig. S2** Representative Oil Red O staining images of leg muscle cross-sections from L1L1 and WW chickens at 6 weeks of age. Red staining indicates neutral lipid accumulation within the intermyofibrillar space. Scale bars = 100 μm. Quantification of Oil Red O staining, expressed as the Oil Red O-positive area fraction, was performed with ImageJ from at least 6 random fields per section in 12 birds per genotype.

**Fig. S3** Representative Oil Red O staining images of breast muscle cross-sections from L1L1 and WW chickens at 6 weeks of age. Red staining indicates neutral lipid accumulation within the intermyofibrillar space. Scale bars = 50 μm. Quantification of Oil Red O staining, expressed as the Oil Red O-positive area fraction, was performed with ImageJ from at least 6 random fields per section in 12 birds per genotype.

**Fig. S4** Validation of overexpression and knockdown efficiencies by qRT-PCR. (A) Relative IGF2BP1 mRNA levels in primary chicken hepatocytes transfected with pcDNA3.1-IGF2BP1 or si-IGF2BP1, with pcDNA3.1 and si-NC as respective controls. (B) Relative CD44 mRNA levels in primary chicken myoblasts transfected with pcDNA3.1-CD44 or si-CD44, with pcDNA3.1 and si-NC as respective controls. Data are presented as means ± SEM (*n* = 4).

**Table S1** Primers used in this study.

| **Table S1 Primers used in this study.** | |
| --- | --- |
| **Primer ID** | **Primer sequence (5'--3')** |
| *IGF2BP1-qF* | *GCCACCATCAGGAACATTACCAA* |
| *IGF2BP1-qR* | *GCACTTCATCAGCTGTCTTCGTG* |
| *IGF2BP1-asp-F* | *CCCCCGTCCCTAGAAACCAGCAAAAC* |
| *IGF2BP1-asp-R* | *GTCTGCTGGGAAGAAGTGGGGGTATATTTG* |
| *VLDLR-qF* | *ATTGGCAGACACAGTGGTTC* |
| *VLDLR-qR* | *GCATATCATCATCTGTGCTT* |
| *LDLR-qF* | *AGGCATCGCGTTGGATCCC* |
| *LDLR-qR* | *CGCTCCAGCAGCACTTTGG* |
| *LPL-qF* | *AGCTGGTCAGACTGGTGGACTC* |
| *LPL-qR* | *TTTACCAAGACGTGAGCTGCCATC* |
| *CD44-qF* | *AGGGACCACACAAGGGAGACG* |
| *CD44-qR* | *GCAGCAACGATGATCAGCCATTC* |
